# Explanations of socioeconomic differences in changes in physical function in older adults: results from the Longitudinal Aging Study Amsterdam

**DOI:** 10.1186/1471-2458-6-244

**Published:** 2006-10-05

**Authors:** Annemarie Koster, Hans Bosma, Marjolein I Broese van Groenou, Gertrudis IJM Kempen, Brenda WJH Penninx, Jacques ThM van Eijk, Dorly JH Deeg

**Affiliations:** 1 Department of Health Care Studies, Section Medical Sociology, Universiteit Maastricht, PO Box 616, 6200 MD Maastricht, The Netherlands; 2 Department of Social-Cultural Sciences, Faculty of Social Sciences, Vrije Universiteit, De Boelelaan 1081, 1081 HV Amsterdam, The Netherlands; 3 Department of Psychiatry, VU University Medical Center, Valeriusplein 91075 BG Amsterdam, The Netherlands; 4 Institute for Research in Extramural Medicine, LASA, VU University Medical Center, Van der Boechorststraat 7, 1081 BT Amsterdam, The Netherlands

## Abstract

**Background:**

This study examines the association between socioeconomic status and changes in physical function in younger- (aged 55–70 years) and older-old (aged 70–85 years) adults and seeks to determine the relative contribution of diseases, behavioral, and psychosocial factors in explaining this association.

**Methods:**

Data were from 2,366 men and women, aged 55–85 years, participating in the Longitudinal Aging Study Amsterdam (LASA). Two indicators of socioeconomic status were used: education and income. Physical function was measured by self-reported physical ability over nine years of follow-up.

**Results:**

In older adults, low socioeconomic status was related to a poorer level of physical function during nine years of follow-up. In subjects who were between 55 and 70 years old, there was an additional significant socioeconomic-differential decline in physical function, while socioeconomic differentials did not further widen in subjects 70 years and older. Behavioral factors, mainly BMI and physical activity, largely explained the socioeconomic differences in physical function in the youngest age group, while psychosocial factors reduced socioeconomic status differences most in the oldest age group.

**Conclusion:**

The findings indicate age-specificity of both the pattern of socioeconomic status differences in function in older persons and the mechanisms underlying these associations.

## Background

Low socioeconomic status (SES) is related to poor physical function and the development of physical disabilities in older adults [1-6]. Disability, usually defined as the inability to carry out the usual tasks of daily living, is established as a powerful measure of health status in old age [7]. In addition, physical disability in older persons is related to many diseases and has been shown to be a predictor of mortality in older adults. Therefore, it is important to understand what factors to intervene upon in order to reduce physical disabilities and the SES differences therein.

Several factors, including biomedical, behavioral, and psychosocial factors, may be important in explaining these SES differences in physical function. Biomedical factors, such as cardiovascular disease, stroke, and diabetes, are related to both low SES and adverse functional outcomes [8-12]. A recent study shows that biomedical factors, including a wide range of diseases and biological risk factors, explained part of the SES differences in mobility limitation incidence in older adults [5]. Low SES is also related to many adverse behavioral factors, such as smoking, excessive alcohol consumption, and decreasing physical activity, that in turn are related to poor functional outcomes [13-16]. Finally, psychosocial factors may be important in explaining SES differences in poor functional outcomes because people with low SES generally have fewer psychosocial resources than people with high SES [17-19]. Psychosocial factors, such as control beliefs and social support, are also linked to ill health and poor functional outcomes [20-22].

The present study examines the association between SES and longitudinal changes in physical function in a representative sample of Dutch older adults, who participated in the Longitudinal Aging Study Amsterdam. Although SES differences in health occur in different age groups, these differences have been less examined in older age groups than in younger age groups. Some studies report that SES differences attenuate in old age [23-26], although evidence is contradictory [27]. The present study gives the opportunity to examine whether the effect of SES on physical function is different for younger-old (aged 55–70 years) and older-old (aged 70–85 years) adults and how these differences develop over nine years of follow-up. The contribution of a number of potential explanatory factors to explain SES differences in physical function has been studied before [5,13,28,29]. In this study, however, we will integrate several explanatory factors, including diseases, behavioral, and psychosocial factors in order to determine the relative contribution of these factors to the explanation of SES inequalities in changes in physical function.

## Methods

### Study sample

Data were from the Longitudinal Aging Study Amsterdam (LASA), an ongoing cohort study on predictors and consequences of changes in well-being and autonomy in older persons in the Netherlands [30]. A random sample of persons, 55–85 years of age, was drawn from the population registers of 11 municipalities. This sample was originally recruited for the NESTOR study on Living Arrangements and Social Networks of older adults (LSN) in 1992 [31]. The response rate was 62.3% (n = 3,805). About eleven months after the LSN interview, 3,107 (81.7%) subjects were enrolled in the main baseline interview of LASA conducted between September 1992 and September 1993. Attrition was significantly associated with age but not with gender. At the first follow-up in 1995/1996, 2,545 participants took part in the study; 416 (13.4%) had died and 146 (4.7%) were lost to follow-up for other reasons (refusal, too frail, could not be contacted). At the second follow-up measurement in 1998/1999, 2,076 persons participated in the study; 343 (13.5%) had died and 126 (5.0%) were lost to follow-up for other reasons. At the third follow-up measurement in 2001/2002, 1,691 persons took part in the study; 289 (13.9%) had died and 96 (4.6%) were lost to follow-up for other reasons. For the present study, respondents were selected with data on physical function at at least two measurements, resulting in 2,366 participants; 1,262 persons aged 55–70 years and 1,104 persons aged 70–85 years. The medical ethical review board of the VU University Medical Center approved the study, and informed consent was obtained from all respondents.

### Measures

#### Socioeconomic status

Two indicators of SES were used, education and income [32]. Categories for education were: high (university, college, higher vocational, general secondary, and intermediate vocational education, ≥11 years) medium (general intermediate, and lower vocational education, 7–11 years), and low (elementary education or less, ≤6 years). For monthly net income, three categories were distinguished: high (greater or equal to 1035 euro), medium (between 625 and less than 1035 euro), and low (less than 625 euro). For participants with a partner living in the household, household income was multiplied by 0.7 to make it comparable to the incomes in a one-person household. Income data at baseline were missing for 342 respondents. For 290 respondents imputations were made using income data collected at the LSN measurement in 1992 or follow-up measurements [3]. For 52 participants income data were missing at all measurements; therefore they were excluded from the analyses with income, resulting in 2,314 participants for the analyses with income.

#### Physical function

Physical function was measured by self-report. At each measurement cycle questions were asked about the degree to which the respondent had difficulty performing six usual daily activities: walking up and down a 15-step staircase without resting, getting (un)dressed, getting up from and sitting down in a chair, cutting own toenails, walking 5 minutes outdoors without resting, and using own or public transport [33]. The response categories were: (0) not able to do, (1) only with help, (2) with much difficulty, (3) with some difficulty, and (4) without difficulty. The sum score of the six items ranged from 0 to 24, with lower scores indicating more limitation in physical function.

#### Covariates

Sociodemographics included age and sex. The presence of diseases at baseline was determined by asking the participants whether they had any of the following chronic diseases: cardiac disease, peripheral arterial disease, stroke, diabetes mellitus, lung disease, cancer, and arthritis [34].

Life-style factors, measured at baseline, included smoking, alcohol use, physical activity, and body mass index (BMI). Four categories of smoking were created: current, former, never and missing. Categories of alcohol use were: not drinking, moderate drinking, excessive drinking (at least three glasses daily), and missing. Physical activity was defined as the total number of physical activities performed in the past two weeks including walking, cycling, gardening, performing light and heavy household, and a maximum of two sports. Four categories were created: 6–7, 4–5, 1–3, and missing. BMI (weight in kilograms divided by height in meters squared) was categorized as lower than 25 kg/m^2^, between 25 and 30 kg/m^2^, greater or equal to 30 kg/m^2^, and missing. Data on behavioral factors were missing because these data were collected in a medical interview in which 86% of the study sample took part.

Psychosocial factors, measured at baseline, included partner status (presence of a partner, yes or no), network size, instrumental social support, emotional social support, mastery, and self-efficacy. The size of the personal network was determined by asking the respondent to identify the persons, other than their partner, whom they had frequent contact with and who were important to them (range is 0–75) [35]. For a maximum of nine network members with whom contact was most frequent, information was collected regarding the intensity of received instrumental social support and social emotional support (range is 0 (no social support)-36). Mastery was measured with a 5-item version of the Pearlin Mastery Scale (range is 5 (lowest)-25) [36]. Self-efficacy was measured with a 12-item version of the General Self-efficacy Scale of Sherer et al. (range is 12 (lowest)-60) [37,38]. Missing values for network size (n = 112), instrumental support (n = 118), emotional support (n = 120), mastery (n = 64), and self-efficacy (n = 107) were replaced by group means in order to maintain an optimal sample size for the analyses.

### Statistical analyses

All analyses were stratified for two age groups; younger than 70 years and 70 years and older. Interaction between SES and age were formally tested. Differences in baseline characteristics between SES groups were determined using chi-square test for categorical variables and t-test statistics for continuous variables. Multilevel analyses, using linear mixed models in SPSS, were used to examine the association between SES and the longitudinal change in physical function. A multilevel analysis has the advantage that it is a suitable technique for repeated measurement analyses. We defined a two-level hierarchy to form random regression models to describe the individual variability in the longitudinal development of physical function. The first level was defined by longitudinal time (in terms of the number of the observation) and the second level by the respondents. Five models were fitted. The first model included SES (education or income) as well as the interaction between SES and longitudinal time to determine how the effect of SES on physical function developed over nine years of follow-up. The model also included sex and age and two interaction terms that were statistically significant; the interaction between age and longitudinal time and between sex and longitudinal time. In model 2, 3, and 4, diseases, behavioral factors, and psychosocial factors were introduced separately into the first model in order to determine the effect of these factors in reducing SES differences in physical function. Interactions between covariates and longitudinal were also tested. The fifth model included all variables of the previous models. Analyses were performed using SPSS version 12.0.

## Results

All analyses were performed in two age groups: younger than 70 years and 70 years and older. We formally tested the Interactions between SES and age; interactions were statistically significant. Interactions between SES and sex were not statistically significant. Table 1 shows baseline characteristics of the total study population by SES and age group. In both age groups there were more women in low SES groups and respondents were older in low SES groups. People with higher education or income had significantly higher baseline physical function scores, indicating better function, compared to low SES groups in both age groups (p < 0.01). There was no strong association between the prevalence of diseases and SES. In the lowest age group, only the prevalence of peripheral atherosclerosis and arthritis was significantly higher in both the lowest education and income groups. In the highest age group, the prevalence of arthritis was also significantly higher in people with a low level of education. In both age groups, there were more non-smokers and fewer nondrinkers in the lowest SES groups (p < 0.01). Persons younger than 70 with high SES were significantly more physically active compared to the lowest SES groups (p < 0.01). In the highest age group this association was present for education, but not for income. In both age groups, people with low SES had a significantly higher BMI compared to people with high SES (p < 0.01). Low SES was also associated with a smaller network, less emotional support, lower feelings of mastery (only in the lowest age group), and lower self-efficacy compared to people with high SES.

Mean physical function scores at the four measurements by age group according to SES are shown in table 2. Low SES groups had worse physical function scores at all measurements compared to high SES groups. The oldest age group had lower physical function scores at all measurements than the youngest age group.

**Figure 1:**
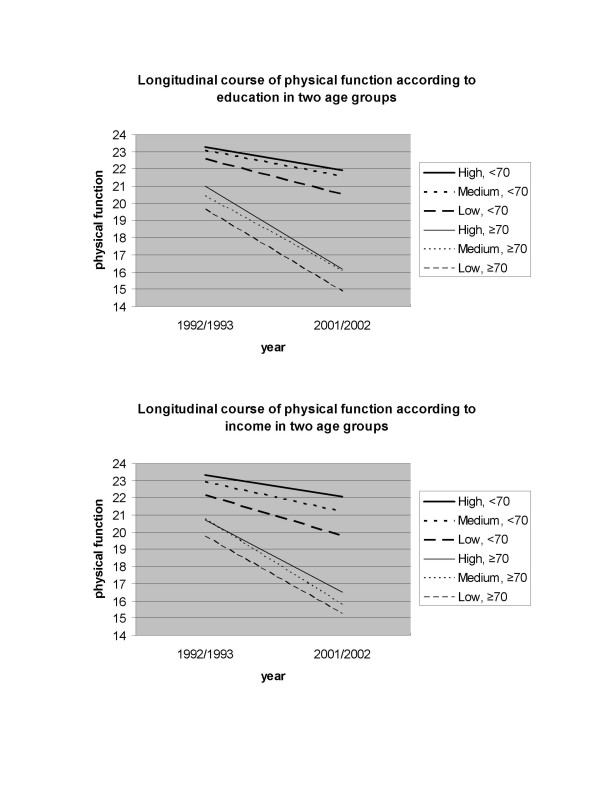
Longitudinal course of physical function* according to socioeconomic status in two age groups (<70 years old and ≥ 70 years old). * Adjusted for age, sex, and interactions between age and longitudinal time, and sex and longitudinal time

Table 3 shows the results of the multilevel analyses for the group younger than 70 years and table 4 for those 70 years and older. The tables present the intercept and slope of the variable time for each model. The intercept is the parameter estimate (baseline score) of the middle and low SES group compared to the highest SES group. The slope is the coefficient of the SES*longitudinal time interaction term, indicating the effect of longitudinal time on physical function. The fist model is graphically presented in figure 1. People younger than 70 years old in the lowest SES group not only had a significantly worse physical function score at baseline and follow-up, they were also declining significantly more over time compared to people in the highest SES group (Table 3: Model 1, Figure 1). Baseline differences between the lowest and the highest education group were larger in the oldest age group. SES differences did, however, not further widen over time in people of 70 years and older (Table 4: Model 1, figure 1).

In model 2, 3, and 4 diseases, behavioral factors, and psychosocial factors were introduced separately into the first model. In the youngest age group (Table 3), the largest reduction in intercepts was found in model 3, due to behavioral factors, for both education and income. A percentage reduction in intercept from model 1 was computed by (intercept _model 1 _- intercept _model 3_)/(intercept _model 1_). The average reduction due to behavioral factors in the lowest SES groups was 65%. BMI and physical activity most reduced the regression coefficients; smoking and drinking did not further decrease the regression coefficients (not tabulated). The average reduction in the lowest SES groups due to diseases was 35% and 26% due to psychosocial factors. None of the explanatory factors could explain the SES-differential decline. The SES differences in baseline physical function were completely explained when all factors of the previous models were added to the fifth model. The differences in slope, however, remained statistically significant.

In the oldest age group, the large differences in baseline physical function between the lowest and the highest SES group could not be fully explained by diseases, behavioral or psychosocial factors (Table 4). The largest reduction in intercept differences between the lowest and the highest SES group in this age group was found in model 4, due to psychosocial factors (on average 28%). In additional analyses, we determined which psychosocial factors were most important in reducing the regression coefficients. None of the psychosocial factors, however, had a large individual contribution. In the full model (Model 5), the intercept of low education still was significantly different from that of higher education.

Because the significant differences in slope between SES groups, that were found in the lowest age group, could not be explained by any of the variables that were added in model 2 to 4, we determined, in additional analyses, whether interactions between each explanatory factor and longitudinal time would reduce the slope differences. However, the coefficients were difficult to interpret which may be due to the complexity and potential over-fitting of the models and, therefore, did not add to the explanation of the slope differences.

## Discussion

In an older population, low SES was associated with poor physical function during nine years of follow-up. In contrast to people 70 years and older, where SES differentials in physical function did not further increase over time, SES differentials in physical function significantly increased over nine years in people who were younger than 70 years. Behavioral factors, mainly BMI and physical activity, explained a large part of the SES differentials in baseline physical function in the youngest age group. Behavioral factors could, however, not explain the SES-differential decline in physical function. In people 70 years and older, psychosocial factors reduced the SES differences most.

An important strength of our study is that we could examine SES differences in physical function in both younger-old and older-old adults. Furthermore, we were able to incorporate a wide range of explanatory factors in the explanation of SES differences in physical function in both age groups. Relatively few studies in older people have investigated the relationship between SES and functional outcomes [1,2,4,5]. Studies have found that health outcomes were weaker in people aged 65 or older compared to younger people. A recent study in women showed that SES inequalities in physical health may attenuate by the age of 70 [24]. This latter study also found a strong cross-sectional association between SES and physical function, but no association with a decline in physical function [24]. The present study shows that even in old age, SES differences in physical function were still present. In people of 70 years and older, SES differences in physical function did not increase over time, but still existed after nine years of follow-up. The lowest SES group probably consists of "healthy survivors" because of selection prior to baseline and attrition due to mortality during the follow-up. It may be due to this healthy survivor effect that SES differentials in physical function did not widen over time in this age group. A recent study showed that mortality selection and cohort effects could not fully explain shrinking educational disparities in functional health in old age and that further research is necessary to explore explanation for diminishing SES differences in health [39].

In the youngest age group, a high percentage had no physical function problems at baseline (not tabulated). The decline in physical function that was found in this age group was mainly due to the onset of physical function problems rather than to a further functional decline related to an already existing disability. This may underlie the age-specificity that was found in both the pattern and the explanation of SES differences in physical function. Behavioral factors, in this study particularly high BMI and low physical activity, may be important in predicting the onset of physical function problems in the youngest age group. Adverse psychosocial factors, such as low social support and low self-efficacy, may cause a further decline in physical function in the oldest age group. Existing physical function problems may negatively influence someone's psychosocial profile, such as lower self-efficacy, which may further accelerate the decline in physical function as in a vicious circle [40]. Such pathways and reciprocal associations need further detailed examination.

In this study, a wide range of different explanatory factors was considered in order to get more insight into the explanation of SES inequalities in physical function. A few drawbacks, however, have to be considered. First, the SES differential decline that was found in the youngest age group could not be explained. This suggests that other factors that were not measured in this study may have caused the widening over time in this age group. Second, the contribution of the explanatory factors in reducing the SES differences and in particular the SES-differential decline in physical function could have been larger if these factors had also been considered longitudinally. However, there is evidence that behavioral factors, mainly smoking and alcohol consumption remain rather stable over time; physical activity showed greater variability over time. These changes in health behavior were, however, not related to SES [41]. Psychosocial factors, such as network size and social support, probably also remained rather stable during the follow-up [35,42]. The prevalence of diseases, however, was more likely to have changed during the nine years of follow-up which may have led to an underestimation of the contribution of these factors. Third, we assumed that the three groups of explanatory factors had an independent contribution to the explanation of SES differences in physical function. The mechanism are probably interrelated, indicating that some mechanisms work through others rather than work independently from each other. This may have had consequences for the estimation of the exact contribution of each group of explanatory factors in the explanation of SES differences in physical function. However, the contribution of diseases, behavioral, and psychosocial factors together is not affected by whether these factors are interrelated or not. Fourth, missing values for psychosocial factors were replaced by group means, which could have led to an attenuation of the effect of these factors in explaining the SES differentials. In additional analyses, however, in which subjects with missing values on psychosocial factors were excluded, the contribution of these factors in the explanation of SES differences in physical function was very similar. Because of a large number of missing data on behavioral factors, a missing category was created and included in the main analyses. The contribution of behavioral factors was also very similar when we excluded subjects with missing data. Finally, we must acknowledge that, had more objective information on diseases been available, the reduction in the strength of the SES effect might have been larger.

Loss to follow-up was a limitation of this study. Attrition was mainly due to mortality (74% of the total loss to follow-up). People who were lost to follow-up for other reasons had worse physical function scores at baseline, compared to those who remained in the study (data not shown). In addition, those who were lost to follow-up also had a significantly lower SES compared to our study population. The association between SES and physical function may therefore have been underestimated. Whether the relative contribution of explanatory variables was equally underestimated remains unknown.

## Conclusion

This study contributes to the understanding of the mechanisms underlying the association between SES and physical function in older adults. In order to reduce physical disabilities in older adults and in particular the socioeconomic differences therein, it may be helpful to improve health-related behaviors in younger-old adults and to intervene upon psychosocial characteristics in older-old adults. Further study is needed to confirm the age-specificity that was found in both the pattern and the explanation of socioeconomic differences in function in older adults.

## Competing interests

The author(s) declare that they have no competing interests.

## Authors' contributions

AK analyzed the data and wrote the first draft of the paper. HB contributed to the conceptualization of the idea, contributed to the data analyses, interpreted the data, and contributed to drafts of the article. MIBG contributed to the conceptualization of the idea, interpreted the data, and reviewed drafts of the article. GIJMK, BWJHP, and JTME interpreted the results and reviewed drafts of the paper. DJHD, scientific director of LASA, interpreted the results and contributed to drafts of the manuscript. All authors approved the final version of the paper.

## Pre-publication history

The pre-publication history for this paper can be accessed here:



## Figures and Tables

**Table 1:** Baseline characteristics of the study population by SES and age group

	< 70 years n = 1262								≥70 years n = 1104							
	
Baseline characteristics	Education				Income				Education				Income			
	
	high n = 401	medium n = 419	Low n = 442	p	high n = 553	medium n = 445	low n = 248	p	high n = 261	medium n = 311	low n = 532	p	high n = 248	medium n = 339	low n = 481	p
Women, %	34.4	54.7	69.9	<0.01	41.0	55.7	75.8	<0.01	40.6	42.4	65.6	<0.01	39.1	41.9	68.2	<0.01
Age, mean (SD)	62.1 (4.2)	62.4 (4.3)	63.2 (4.2)	<0.01	61.5 (4.0)	62.9 (4.2)	64.4 (4.2)	<0.01	77.4 (4.3)	76.7 (4.2)	78.0 (4.3)	<0.01	77.4 (4.2)	76.9 (4.3)	77.7 (4.3)	0.03
Physical function																
Baseline score (0–24), mean (SD)	23.4 (2.1)	23.1 (2.4)	22.4 (3.4)	<0.01	23.4 (1.8)	22.9 (2.7)	21.8 (4.0)	<0.01	21.3 (4.2)	20.8 (4.7)	19.1 (5.6)	<0.01	20.9 (4.3)	21.2 (4.3)	19.1 (5.8)	<0.01
*Diseases*																
Cardiac disease, %	13.5	15.3	12.2	0.42	13.4	14.0	14.1	0.95	23.1	25.2	23.8	0.83	24.3	24.9	23.9	0.95
Peripheral atherosclerosis, %	5.0	4.8	9.7	<0.01	3.1	9.2	10.1	<0.01	11.5	12.3	10.1	0.60	8.9	10.7	12.2	0.41
Stroke, %	1.0	2.6	2.5	0.19	1.3	2.0	4.0	0.04	5.8	6.8	6.8	0.84	5.7	6.5	6.7	0.86
Diabetes mellitus, %	3.5	3.8	4.8	0.63	3.4	5.4	3.2	0.22	5.8	6.8	8.6	0.33	6.5	6.2	8.4	0.44
Lung disease, %	7.5	7.2	10.6	0.13	7.2	9.2	10.5	0.27	13.1	13.2	12.5	0.95	14.6	11.9	12.8	0.63
Cancer, %	5.0	7.0	8.1	0.19	6.3	6.3	8.9	0.36	6.7	11.3	11.6	0.11	8.1	10.7	11.3	0.39
Arthritis, %	26.3	29.9	35.7	0.01	25.0	34.0	38.7	<0.01	33.8	31.3	40.3	0.02	34.4	32.6	40.0	0.07
*Behavioral factors*																
Smoking, %																
non-smoker	15.5	26.5	32.4	<0.01	19.2	26.1	35.1	<0.01	23.4	26.7	39.3	<0.01	22.2	25.4	42.2	<0.01
former	49.9	41.5	33.3		46.5	40.2	32.3		45.2	45.0	28.9		50.0	42.5	28.5	
smoker	28.4	23.2	25.3		28.2	24.9	21.4		18.8	19.0	15.4		15.3	20.4	16.0	
missing	6.2	8.8	9.0		6.1	8.8	11.3		12.6	9.3	16.4		12.5	11.8	13.3	
Dinking, %																
non-drinker	9.0	13.4	23.1	<0.01	11.2	17.5	21.0	<0.01	15.3	19.0	26.1	<0.01	27.9	17.7	14.9	<0.01
moderate	77.1	73.0	64.3		75.6	68.3	66.1		67.8	67.5	55.8		56.5	67.8	67.7	
excessive	7.7	4.5	3.4		6.7	5.4	1.6		3.4	3.5	1.3		1.9	2.4	3.6	
missing	6.2	9.1	9.3		6.5	8.8	11.3		13.4	10.0	16.7		13.7	12.1	13.7	
Physical activity, %																
6–7	29.9	28.6	20.1	<0.01	28.9	23.4	25.0	<0.01	11.5	11.9	9.2	<0.01	10.9	10.3	11.0	0.83
4–5	45.6	44.9	44.1		46.5	47.6	37.1		33.3	41.2	39.8		37.5	41.9	39.3	
0–3	22.2	19.6	29.6		21.9	22.0	31.5		48.3	42.1	39.7		46.0	42.5	42.4	
missing	2.2	6.9	6.1		2.7	7.0	6.5		6.9	4.8	11.3		5.6	5.3	7.3	
BMI, %																
<25	38.2	31.7	23.1	<0.01	35.6	27.6	26.2	<0.01	41.4	29.6	21.2	<0.01	34.3	31.0	23.3	<0.01
25-<30	42.9	44.4	47.3		47.4	43.8	40.7		33.3	44.7	34.8		33.1	42.5	37.4	
≥30	10.7	14.1	18.6		8.9	18.0	21.0		10.0	14.8	24.2		16.9	12.4	23.5	
missing	8.2	9.8	11.1		8.1	10.6	12.1		15.3	10.9	19.7		15.7	14.2	15.8	
*Psychosocial factors*																
With partner, %	84.0	81.1	75.3	<0.01	86.8	82.2	60.5	<0.01	62.1	65.3	45.1	<0.01	62.1	65.5	45.3	<0.01
Network size (0–75), mean (SD)	16.4 (9.3)	15.3 (8.3)	14.2 (7.9)	<0.01	16.3 (9.1)	14.8 (7.8)	13.9 (8.4)	<0.01	14.3 (8.0)	15.5 (8.2)	12.2 (6.8)	<0.01	14.6 (8.2)	13.4 (7.8)	12.0 (7.1)	<0.01
Instrumental support (0–36), mean (SD)	14.8 (6.5)	14.4 (6.1)	14.1 (6.7)	0.36	14.4 (6.1)	14.3 (6.6)	14.9 (7.0)	0.49	14.0 (6.8)	13.9 (6.8)	14.4 (6.8)	0.44	13.9 (6.6)	13.9 (6.4)	14.5 (7.4)	0.33
Emotional support (0–36), mean (SD)	23.4 (7.3)	22.9 (7.2)	22.2 (7.9)	0.07	23.1 (7.3)	22.7 (7.6)	22.4 (7.4)	0.48	22.5 (8.0)	21.0 (8.1)	19.9 (7.8)	<0.01	22.0 (8.0)	21.0 (7.8)	20.1 (8.4)	0.02
Mastery (5–25), mean (SD)	18.1 (3.2)	17.9 (3.1)	17.5 (3.4)	0.03	18.2 (3.1)	17.8 (3.2)	17.2 (3.3)	<0.01	17.0 (3.1)	17.2 (3.2)	16.7 (3.2)	0.08	16.9 (3.1)	16.9 (3.3)	16.9 (3.1)	0.99
Self-efficacy (12–60), mean (SD)	44.0 (5.1)	42.9 (4.8)	41.3 (5.5)	<0.01	43.4 (5.2)	42.5 (5.2)	41.4 (5.4)	<0.01	42.4 (5.3)	41.5 (5.0)	40.6 (4.8)	<0.01	42.1 (5.0)	41.4 (5.0)	40.8 (5.1)	<0.01

**Table 2:** Mean physical function score * at 4 measurements by age group according to SES

	<70 years								≥70 years							
	
	1992/1993		1995/1996		1998/1999		2001/2001		1992/1993		1995/1996		1998/1999		2001/2001	
	n	Mean (SD)	n	Mean (SD)	N	Mean (SD)	n	Mean (SD)	N	Mean (SD)	n	Mean (SD)	n	Mean (SD)	n	Mean (SD)
Education																
High	400	23.4 (2.1)	389	23.0 (2.6)	363	22.7 (3.0)	329	22.3 (3.2)	261	21.3 (4.2)	257	19.9 (5.1)	173	18.9 (6.0)	113	18.8 (5.8)
Medium	419	23.1 (2.4)	412	22.5 (3.3)	370	22.5 (3.2)	323	21.8 (3.7)	310	20.8 (4.7)	305	19.8 (5.6)	233	18.8 (5.5)	145	18.7 (5.6)
Low	442	22.4 (3.4)	425	21.9 (3.7)	388	21.2 (4.4)	339	20.6 (4.6)	531	19.1 (5.6)	510	17.8 (6.1)	345	16.6 (6.5)	219	15.7 (6.4)
Overall	1261	22.9 (2.7)	1226	22.5 (3.3)	1121	22.1 (3.6)	991	21.6 (3.9)	1102	20.1(5.2)	1072	18.8 (5.8)	751	17.8 (6.2)	477	17.4 (6.2)
Income																
High	552	23.4 (1.8)	543	23.1 (2.4)	499	22.9 (2.6)	457	22.4 (2.9)	248	20.9 (4.3)	246	19.9 (5.1)	177	18.8 (5.8)	113	18.8 (5.9)
Medium	445	22.9 (2.7)	432	22.4 (3.1)	394	21.9 (3.6)	355	21.3 (4.1)	339	21.2 (4.3)	326	19.6 (5.4)	247	18.2 (6.3)	157	18.0 (6.0)
Low	248	21.8 (4.0)	236	21.3 (4.7)	218	20.6 (5.0)	176	19.8 (5.2)	479	19.1 (5.8)	465	18.0 (6.1)	318	17.2 (6.1)	204	16.2 (6.1)
Overall	1245	22.9 (2.8)	1211	22.5 (3.3)	1111	22.1 (3.6)	988	21.6 (4.0)	1066	20.2 (5.1)	1037	19.0 (5.7)	742	17.9 (6.1)	474	17.4 (6.1)

* Score ranged from 0 to 24, higher scores indicate better physical ability

**Table 3:** Intercept and slope of SES on physical function in group <70 years old

	Model 1 * sociodemographics				Model 2 † model 1+ diseases				Model 3 ‡ model 1+ behavioral factors				Model 4 § model 1+ psychosocial factors				Model 5 ¶ all variables previous models			
	
	Intercept	p	Slope	p	Intercept	p	Slope	p	Intercept	p	Slope	p	Intercept	p	Slope	p	Intercept	p	Slope	p
Education																				
High	Ref.																			
Medium	-0.16	0.45	-0.04	0.59	-0.08	0.69	-0.04	0.62	-0.07	0.73	-0.04	0.59	-0.14	0.49	-0.04	0.59	0.01	0.98	-0.04	0.62
Low	-0.43	0.05	-0.24	<0.01	-0.24	0.26	-0.24	<0.01	-0.07	0.74	-0.24	<0.01	-0.35	0.11	-0.24	<0.01	0.07	0.73	-0.24	<0.01
Income																				
High	Ref.																			
Medium	-0.22	0.26	-0.17	0.02	-0.05	0.79	-0.17	0.03	0.03	0.87	-0.17	0.02	-0.15	0.44	-0.17	0.02	0.18	0.35	-0.16	0.03
Low	-0.83	<0.01	-0.36	<0.01	-0.62	0.01	-0.36	<0.01	-0.46	0.06	-0.36	<0.01	-0.56	0.02	-0.36	<0.01	-0.16	0.50	-0.35	<0.01

* Model 1: adjusted for age, sex, and interactions between age and longitudinal time, and sex and longitudinal time

† Model 2: adjusted for age, sex, interactions between age and longitudinal time and sex and longitudinal time, cardiac disease, peripheral arterial disease, stroke, diabetes mellitus, lung disease, cancer, arthritis

‡ Model 3: adjusted for age, sex, interactions between age and longitudinal time and sex and longitudinal time, smoking, drinking, BMI, and physical activity

§Model 4: adjusted for age, sex, interactions between age and longitudinal time and sex and longitudinal time, partner status, network size, instrumental social support, emotional social support, mastery, and self-efficacy

¶Model 5: all variables of previous models

**Table 4:** Intercept and slope of SES on physical function in group ≥70 years old

	Model 1 * sociodemographics				Model 2 † model 1+ diseases				Model 3 ‡ model 1+ behavioral factors				Model 4 § model 1+ psychosocial factors				Model 5 ¶ all variables previous models			
	
	Intercept	p	Slope	p	Intercept	p	Slope	p	Intercept	p	Slope	p	Intercept	p	Slope	p	Intercept	p	Slope	p
Education																				
High	Ref.																			
Medium	-0.74	0.12	0.16	0.39	-0.66	0.15	0.15	0.41	-0.76	0.10	0.16	0.39	-0.69	0.14	0.16	0.39	-0.68	0.12	0.15	0.41
Low	-1.36	<0.01	0.01	0.94	-1.17	<0.01	-0.00	0.99	-1.16	<0.01	0.01	0.96	-1.04	0.02	0.01	0.95	-0.83	0.04	-0.01	0.97
Income																				
High	Ref.																			
Medium	0.30	0.52	-0.26	0.16	0.29	0.52	-0.25	0.17	0.18	0.68	-0.26	0.16	0.41	0.38	-0.26	0.15	0.22	0.60	-0.25	0.17
Low	-0.86	0.06	-0.10	0.57	-0.67	0.12	-0.13	0.47	-0.80	0.06	-0.10	0.58	-0.59	0.19	-0.10	0.56	-0.47	0.26	-0.12	0.48

* Model 1: adjusted for age, sex, and interactions between age and longitudinal time, and sex and longitudinal time

† Model 2: adjusted for age, sex, interactions between age and longitudinal time and sex and longitudinal time, cardiac disease, peripheral arterial disease, stroke, diabetes mellitus, lung disease, cancer, arthritis

‡ Model 3: adjusted for age, sex, interactions between age and longitudinal time and sex and longitudinal time, smoking, drinking, BMI, and physical activity

§Model 4: adjusted for age, sex, interactions between age and longitudinal time and sex and longitudinal time, partner status, network size, instrumental social support, emotional social support, mastery, and self-efficacy

¶Model 5: all variables of previous models
